# Supplementary material for: A Special Phenotype of Aconidial Aspergillus niger SH2 and Its Mechanism of Formation via CRISPRi
Source: J Fungi (Basel). 2022 Jun 28;8(7):679. doi: 10.3390/jof8070679 (PMC9319794; doi:10.3390/jof8070679)
Supplement: Supplementary file 1 [file jof-08-00679-s001.zip › Supplemental information-1.pdf]

Supplementary information for

Spore-like Propagule: A Special Phenotype in Aconidial *Aspergillus niger* SH2 and Its Mechanism of Formation via CRISPRi

Le-Yi Yu, Lin-Xiang Li, Lin-Lin Yao, Jun-Wei Zheng, Bin Wang, Li Pan*

School of Biology and Biological Engineering, South China University of Technology, Guangzhou Higher Education Mega Center, Guangzhou 510006, China; 995495986@qq.com (L.Y.Y.); 329958317@qq.com (L.X.L.); linlinyaozsyx@163.com (L.L.Y.); 1076241382@qq.com (J.W.Z.); btbinwang@scut.edu.cn (B.W.);

* Correspondence: btlipan@scut.edu.cn (L.P.)

Supplementary materials include the following:

Total pages: 16; total figures: 4; total tables: 12;

Table S1 The strains used in this study.

Table S2 The semi-quantitative RT-PCR system.

Table S3 The primers used in this study.

Table S4 The sequences of the d*Cas9-Mxi1* ORF, common sgRNA cassette and *pyrG* sgRNA expression cassette.

Table S5 The T7 transcription system of the protospacer RNA.

Table S6 The ethanol precipitation system of the RNA.

Table S7 The NLS-Cas9 Nuclease cleavage of the target gene system in vitro.

Table S8 The quality control of the RNA samples.

Table S9 The expression of conidia-associated genes (CAGs) in the spore-like propagule and normal hypha.

Table S10 The expression of germination-associated genes (GeAGs) in the spore-like propagule and normal hypha.

Table S11 The expression of genes involved in the production of enzymes involved in cell wall synthesis or processing.

Table S12 The expression and spacer RNA sites of genes involved in the N-acetyl-D-glucosamine metabolic pathway and conidia.

Figure S1 The flowchart of spore-like propagule purification.

Figure S2 The RNA sequence of spore-like propagule.

Figure S3 The flow chart of CRISPRi construction on conidia and GlcNAc metabolic pathway genes.

Figure S4 The construction of CRISPRi on conidia and GlcNAc metabolic pathway genes in the *Aspergillus niger* SH2.

Table S1 The strains and plasmids used in this study.

Strain / Plasmid	Genotype / Marker	Source
E. coli Mach1™ T1	Host of Molecular cloning	Invitrogen, USA
A. niger SH2	Wild type	A factory, China
A. niger CBS513.88	Wild type	CBS-KNAW, Netherlands
A. niger SH2 Δ*ku*Δ*pyrG*	Δ*ku*Δ*pyrG*	This study
A. niger SH2 Δ*Dac1*	Δ*ku*Δ*pyrG*Δ*Dac1*::*pyrG*	This study
A. niger SH2 *Dac1* recovery	Δ*ku*Δ*pyrG*	This study
A. niger SH2 CRISPRi-*Dac1*	Δ*ku*Δ*pyrG* dCas9-Mxi1 ORF-AMA1:*pyrG*; *Dac1*-sgRNA ORF:*hygB*	This study
A. niger SH2 CRISPRi-*Uap1*	Δ*ku*Δ*pyrG* dCas9-Mxi1 ORF-AMA1:*pyrG*; *Uap1*-sgRNA ORF:*hygB*	This study
A. niger SH2 CRISPRi-*Ngt1*	Δ*ku*Δ*pyrG* dCas9-Mxi1 ORF-AMA1:*pyrG*; Ngt*1*-sgRNA ORF:*hygB*	This study
A. niger SH2 CRISPRi-*Hxk1*	Δ*ku*Δ*pyrG* dCas9-Mxi1 ORF-AMA1:*pyrG*; *Hxk1*-sgRNA ORF:*hygB*	This study
A. niger SH2 CRISPRi-*hfbD*	Δ*ku*Δ*pyrG* dCas9-Mxi1 ORF-AMA1:*pyrG*; *hfbD*-sgRNA ORF:*hygB*	This study
A. niger SH2 CRISPRi-GC	Δ*ku*Δ*pyrG* dCas9-Mxi1 ORF-AMA1:*pyrG*; GC-sgRNA ORF:*hygB*	This study
A. niger SH2 CRISPRi-MAPK1	Δ*ku*Δ*pyrG* dCas9-Mxi1 ORF-AMA1:*pyrG*; MAPK1-sgRNA ORF:*hygB*	This study
A. niger SH2 CRISPRi-d*Cas9-Mxi1*	Δ*ku*Δ*pyrG* dCas9-Mxi1 ORF-AMA1:*pyrG*	This study
A. niger SH2 CRISPRi-d*Cas9*	Δ*ku*Δ*pyrG* dCas9 ORF-AMA1:*pyrG*	This study
A. niger SH2 CRISPRi-non spacer RNA	Δ*ku*Δ*pyrG* dCas9-Mxi1 ORF-AMA1:*pyrG*; non spacer -sgRNA ORF:*hygB*	This study

Table S2 The semi-quantitative RT-PCR system.

Green taq mix (Vazyme, China)	10 μL
cDNA	1 μL
Primer-F	1 μL
Primer-R	1 μL
ddH_2O	7 μL
Total	20 μL

Cycles: 27

Table S3 The primers used in this study.

Primer name	Primer sequences (5'→3')
**Primers used for qPCR**	
Dac1-qPCR-F	GACGGGTGTGACTTCATACT
Dac1-qPCR-R	AGTGAGGTTCTCTTTGCCATA
Uap1-qPCR-F	TTTCCAGCAGCACAACTACTT
Uap1-qPCR-R	AGTAGGTGTGAATGTGCTGG
DC-qPCR-F	CACTCATATCCTTGCCGTTC
DC-qPCR-R	ATTCCATTAGTCTCGCAGCAA
GC-qPCR-F	GAAACTTCGCCAACCGCTC
GC-qPCR-R	TCCTCAGGTTCATCTTCATCA
hxkA-qPCR-F	CCTACCCCGCCACCCAG
hxkA-qPCR-R	GTGTAAGAAGAGGCGATGAG
Ngt1-qPCR-F	ACCTGGGTGGCGTGATTGG
Ngt1-qPCR-R	GGTGTCGTAGAGACCTTTGA
MAPK1-qPCR-F	ATCCCGACGATAGACAGAGA
MAPK1-qPCR-R	GACATTGCCTCGTCGCTCA

Table S4 The sequences of the d*Cas9-Mxi1* ORF, common sgRNA cassette and *pyrG* sgRNA expression cassette.

1. The sequences of the d*Cas9-Mxi1* ORF

Green: *Ptef*
Black: linker
Orange: SV40 NLS
Blue: d*Cas9*
Purple: *Mxi1*
Red: Ttef

cgagacagcagaatcaccgcccaagttaagcctttgtgctgatcatgctctcgaacgggccaagttcgggaaaagcaaaggagcgtttagtg
aggggcaatttgactcacctcccaggcaacagatgagggggcaaaaagaaagaaattttcgtgagtcaatatggattccgagcatcattttct
tgcggtctatcttgctacgtatgttgatcttgacgctgtggatcaagcaacgccactcgctcgctccatcgcaggctggtcgcagacaaattaaa
aggcggcaaactcgtacagccgcggggttgtccgctgcaaagtacagagtgataaaagccgccatgcgaccatcaacgcgttgatgccca
gctttttcgatccgagaatccaccgtagaggcgatagcaagtaaagaaaagctaaacaaaaaaaaatttctgcccctaagccatgaaaacgag
atggggtggagcagaaccaaggaaagagtcgcgctgggctgccgttccggaaggtgttgtaaaggctcgacgcccaaggtgggagtctag
gagaagaatttgcatcgggagtggggcgggttacccctccatatccaatgacagatatctaccagccaagggtttgagcccgcccgcttagtc
gtcgtcctcgcttgcccctccataaaaggatttcccctcccccctcccacaaattttctttcccttcctctccttgtccgcttcagtacgtatatcttcc
cttccctcgcttctctcctccatccttctttcatccatctcctgctaacttctctgctcagcacctctacgcattactagccgtagtatctgagcacttct
ccttttatattccacaaaacataacacaaccttcaccggccattacccaagaaaaagcgcaaggtgGGGCCCatggacaagaagtatag
catcgggctggctattggaacgaactcggttggttgggctgtgattacggacgaatacaaggtgccatccaagaagtttaaggtcctgggaaa
caccgaccgtcactcaatcaagaagaatctcattggagccctgctcttcgatagtggggagaccgccgaagctactcgactgaagcgaacgg
ctcgccggcgttatacacgacgcaagaatcgcatctgctacctccaggagattttcagcaacgaaatggctaaggttgatgactcattctttcat
cgactcgaagaaagtttcttggtcgaggaggataagaagcacgagcgccatccgatctttggtaacattgtggatgaggttgcctatcacgaa
aagtacccaactatctatcatcttcgtaagaagctggtcgatagcacggacaaggctgatttgcgacttatctacctggcactcgcgcacatgat
taagttccgcggccattttcttatcgagggtgacctgaaccccgataattctgacgttgataagctcttcatccagttggtccaaacctacaatcag
ctgtttgaggaaaaccctattaatgcatctggcgtggacgccaaggctatcctttcggcgcgcctgtctaagtcgcggcgtttggagaaccttat
cgcacaactccccggcgaaaagaagaacggcctcttcggtaatttgattgcgttgtcacttggtctgactcctaacttcaagagtaattttgacct
ggcagaggatgcgaagctccagttgtctaaggatacgtatgatgacgatctcgacaacttgcttgcccaaatcggtgaccagtacgctgatctt
ttcctggccgctaagaatctctcagatgcaatcctgctcagtgacattttgcgggtcaacaccgagattactaaggccccccctgtcagctagtat
gatcaagcggtatgatgagcaccatcaggacctcaccttgcttaaggccctcgtgcgtcagcaattgcctgagaagtacaaggaaatcttcttt
gaccaatccaagaacggatacgcagggtatattgatggcggtgcgagccaggaggaattctacaagtttatcaagccgattttggagaagatg
gacggcactgaggaactgctcgtcaagctgaatcgcgaagatttgcttcgtaagcaacgaacgttcgacaacggctccatcccgcaccagat
tcatctgggcgagctccacgccatccttcgacgccaggaagatttctacccatttctgaaggacaaccgtgagaagatcgaaaagattcttaca
ttccgaatcccctactatgtgggacctttggcccgtgggaattcccgatttgcttggatgacccgaaagagcgaggaaaccatcactccgtgga
acttcgaggaagtcgtggacaagggtgcatccgcgcagagcttcattgagcggatgaccaattttgataagaaccttccgaatgaaaaggtcc
tgccaaagcattcgctgctctacgagtatttcaccgtgtataacgaactgactaaggtcaagtacgtgacggagggaatgcggaagccagcct
tcctctcaggggaacaaaagaaggctatcgtcgatttgctttttaagaccaatcgtaaagtgactgttaagcagctgaaggaggattatttcaag
aagattgaatgtttcgactccgtcgagatcagcggcgtggaagatcgctttaacgcttccctcggtacctaccacgacctgctcaagatcattaa
ggacaaggatttcctcgataacgaggaaaatgaggacatcttggaagatattgtcctcacgttgacactttttgaggaccgcgaaatgatcgag
gaacggctcaagacatatgcccatttgttcgacgataaggtgatgaagcagctgaagcggcgtcgatacaccggatggggtcgccttagccg
gaagctgatcaacggcattcgagataagcaatctggtaagactatcttggatttccttaagtcggacggcttcgccaaccgcaattttatgcagct
tattcacgacgattccctgacgttcaaggaggacatccagaaggcacaagtctcaggacaaggggattccctgcacgagcatatcgccaacc

tggctggatccccggcgatcaagaaggggattcttcagaccgtcaaggttgtcgacgagctggtcaaggtgatgggccgtcataagccaga
aaacatcgtgattgagatggcccgagaaaatcagaccactcaaaagggtcagaagaacagccgcgagcggatgaagcggatcgaggaag
gcattaaggaacttggttctcagatcctgaaggagcaccctgttgaaaacacacagctccaaaatgagaagctgtatctctactatttgcaaaat
ggacgcgacatgtacgtcgatcaggagctcgacattaaccggttgtcggactacgatgttgacgctatcgtcccgcaatccttccttaaggacg
atagcattgataacaaggtgctgactcgctcagataagaaccggggcaagtccgacaatgttccaagcgaggaagtggttaagaagatgaag
aactactggcgccaattgcttaatgccaagctcatcacacagcgcaagtttgacaacttgaccaaggccgagcggggagggctgagtgaact
cgataaggctggcttcatcaagcgtcaactcgtggagacgcgacagatcacaaagcacgttgctcagattctggactcccggatgaacacaa
agtacgacgagaatgataagctcatccgtgaagttaaggtcattaccctcaagtctaagttggtgtcggatttccgcaaggacttccaattttata
aggttcgggagatcaacaattatcaccatgcacatgatgcgtacctcaacgcagtcgtgggaactgcgctcatcaagaagtatcccaagttgg
agtccgaattcgtctacggggattataaggtttacgacgtccgcaagatgatcgccaagagtgagcaggaaattggcaaggccacggctaag
tatttcttttactccaacatcatgaatttctttaagacggagatcacactcgccaatggagaaatccgtaagcgacctttgattgagaccaacggc
gagactggtgaaatcgtttgggataaggggcgcgacttcgctaccgtgcggaaggttctgagcatgccgcaagtcaatatcgtcaagaaaac
cgaggtgcagacaggcggtttctctaaggaatcgattcttccaaagcgtaactctgacaagctgatcgctcgaaagaaggattgggaccccaa
gaagtatggagggttcgattctcctacagtggcatactcggttctcgttgtcgcgaaggttgagaagggaaagtctaagaagctgaagtcggtc
aaggaactgctcgggatcaccattatggagcgctccagcttcgaaaagaatcccatcgactttctcgaggccaagggctataaggaagtcaa
gaaggatcttatcattaagctgcctaagtactctttgttcgagcttgaaaacggtcgaaagcgaatgctcgcatcggcaggagagttgcagaag
gggaatgaattggcacttccctcaaagtacgtgaacttcctgtatctcgcgtcccactacgagaagctgaagggtagccctgaggacaacgaa
cagaagcaactttttgttgagcaacacaagcattatctggatgagatcattgaacagatttcagagttcagtaagcgcgtcatcctcgccgatgct
aatctcgacaaggtgttgtcggcctacaacaagcaccgtgacaagccgatccgagagcaggctgaaaatatcattcatctgttcaccctcacta
acttgggagcaccagcagcgttcaagtattttgatacgacaatcgaccgtaagcgatacacgtccacaaaggaggtgcttgatgcgaccctga
ttcatcaatccatcactgggctctatgaaacccgtatcgaccttagtcaactgggggcgacGGGCCCcccaagaaaaagcgcaaggtg
ggtagttccaagcttggcggcagcggcggcagcatggaacgtgtgagaatgattaatgtgcaaaggctgttagaagccgcagagtttttaga
aagaagagaaagagaatgcgaacacgggtatgccagttctttccctagcatgccctctcccagaggctaacctcggccgcggacattcgattt
atgccgttatgacttccttaaaaaagcctttacgaatgaaagaaatggaattagacttgttatgtagttgattctacaatggattatgattcctgaact
tcaaatccgctgttcattattaatctcagctcttcccgtaaagccaatgttgaaactattcgtaaatgtacctcgttttgcgtgtaccttgcttatcacgt
gatattacatgacctggacagagttctgcgcgaaagtcataacgtaaatcccgggcggtaggtgcgtcccgggcggaaggtagttttctcgtc
caccccaacgcgtttatcaacctcaactttcaacaaccatcatgccaccaaaagcgcgtaaaacaaagcgagatttgattgagcaagagggca
ggatccaatgcgcgattcaagacattaaaaatggaaaatttcaaaaaattgcgcccgcagcgcgtgcatacaaaattcatcccaatac

2. The sequences of the common sgRNA cassette

Light green: *aamA* upstream homologous arm

Green: *A. fumigatus* U6 promoter

Black: linker

Orange: *Not I* restriction enzyme cutting site

Blue: sgRNA scaffold

Red: *A. oryzae* U6 terminator

Purple: *hygB* cassette

Wathet: *aamA* downstream homologous arm

TGATAGTATCCGAAAGCTGCAAATTGCTTCATCGAGGCTGGCATTCGATAGAAGAAAG
AATTATAGACAACTAGTCTTGCAATATGACAATTCTCTTTGATTAATAAATGAAAGCACG
CATGTATCAGCCTAATAGCCGAGTGGCGGGCATCTCTGGCGGCCTCCCGAGCAGCGTG
GAATGCGTCCAAGATCCCGTCCGCGGGTCGTCCTTCGGTCGGAATGATGACTGGAGCA
GCAGACGATGTCCTGAGCTGAATGCATGTGATATTCACATTCCAGGGAGAATTGTCGGC
TATTTAGAACCCTCTCGGCTTAAAAGCCCTATTAGACTATGGGTGCGCTCAAGCCACTA
GCCAGGATATCCCGCTGAACGCTCCATCACCTTGCAGCTGAAGTGCAACATGGACGG

GCTTTAACTTTTCGTAGATATAAGTTTAATTTATCCTCTCCACACCCATAGGGTCGTATGG
TGTCAACCGGTGTAGTCTGCAGGATTTCATCTCGCTTCGCCAAGCGAGGCGCCCTAAC
GGGCAGCCTGCAGCTTACCCTGTTAACCCCGGCTCACCACCCCCCGAGCAATCCGTCG
CGTCCTCCACGAGTCATAACAAGGTTCGGGCGTTGTTTCTTACCCCCACTATCAGGCGT
ATTCAGTTAACAGTCAGTAGTCCCGTGTCGGAGATTTGTTGTTCTGCAACAATTAAAGG
GGACCAGGGTTAAATCCTGGCCCCCGAACTGATCGGAGTTTCGGCCAATGAGAGATGT
TGTATACCCCCGTTCCTGGCAGATGGATTAATTGCCGGCTCCATTTGGCATCCATCAAGC
ATCATACGGGATTAGAAGGGTAGTTCGTGGGTTGATCTGCCGTGCAAGGTGCTCAAGG
CTCTGGAGTCATGCTGAACGCAAATATTTAAGAATCGTCGTCAGGGACAGCGTTCTCTG
GATAGTCAAGCTGTGCTTGGGACGCTGTTCTGTCGCTTTGTCAAAACATAATTCGCAGC
GGAATTCTTAATTAAgcaggcggttgcaagcgatctatcgtgaatttgtggagggacgtaatggtgaaatgccgaatggcgaag
caggtcgggagatcggtggtcggacgagcctgatgtagatgggtgcatatctttcttttctctttcacaatttcaagatcatgtattagaactacatt
gctatgtttcagcaatatatatgcatagctagagagcgatgttacgatgagacatgtaatctttgaacatttctctgtacagcttgtcagagtttcga
aggttaagctcgagagcaaggaggggaaaagaagacaaaaaagacactcggtccggtctcgaaccgacgacctttcgcacacacgtagaa
ggtacagaaactcaacacctccaaaagaggtctgtaagaatgaaaGCGGCCGCgttttagagctagaaatagcaagttaaaataaggc
tagtccgttatcaacttgaaaaagtggcaccgagtcggtgcttttttttgagcatttatcagcttgatatagaggtaggaatgtatggaggtgcag
aatggctattttgttattggagcgggttcgaaacggagggcaggagactttttctaaatacgtcacgtgatatagagctgctGGGCCCGG
TACCCGGGGATCCgctagtggaggtcaacacatcaatgctattttggtttagtcgtccaggcggatcacaaatttgtgtcgtttgaca
agatggttcatttaggcaactggtcagatcagcccacttgtaagcagtagcggcggcgctcgaagtgtgactcttattagcagacaggaacga
ggacattattatcatctgctgcttggtgcacgataacttgtgcgtttgtcaagcaaggtaagtgaacgacccggtcataccttcttaagttcgccct
tcctccctttatttcagattcaatctgacttacctattctacccaagcatccaaatgcctgaactcaccgcgacgtctgtcgagaagtttctgatcga
aaagttcgacagcgtctccgacctgatgcagctctcggagggcgaagaatctcgtgctttcagcttcgatgtaggagggcgtggatatgtcct
gcgggtaaatagctgcgccgatggtttctacaaagatcgttatgtttatcggcactttgcatcggccgcgctcccgattccggaagtgcttgaca
ttggggagttcagcgagagcctgacctattgcatctcccgccgtgcacagggtgtcacgttgcaagacctgcctgaaaccgaactgcccgct
gttctggagccggtcgcggaggccatggatgcgattgctgcggccgatcttagccagacgagcgggttcggcccattcggaccgcaagga
atcggtcaatacactacatggcgtgatttcatatgcgcgattgctgatccccatgtgtatcactggcaaactgtgatggacgacaccgtcagtgc
gtccgtcgcgcaggctctcgatgagctgatgctttgggccgaggactgccccgaagtccggcacctcgtgcacgcggatttcggctccaaca
atgtcctgacggacaatggccgcataacagcggtcattgactggagcgaggcgatgttcggggattcccaatacgaggtcgccaacatcttct
tctggaggccgtggttggcttgtatggagcagcagacgcgctacttcgagcggaggcatccggagcttgcaggatcgccgcgcctccgggc
gtatatgctccgcattggtcttgaccaactctatcagagcttggttgacggcaatttcgatgatgcagcttgggcgcagggtcgatgcgacgca
atcgtccgatccggagccgggactgtcgggcgtacacaaatcgcccgcagaagcgcggccgtctggaccgatggctgtgtagaagtactc
gccgatagtggaaaccgacgccccagcactcgtccgagggcaaaggaatagagtagatgccgaccgCCAGACTTCTTCCCG
GGCTACCAGGAAGCAGCAGGTGTCTACTGCGTCGGCGAAGTCGACAACGGCAACCCT
GCCCTCGACTGCCCATACCAGAAGGTCCTGGACGGCGTCCTCAACTATCCGATGTACAT
CCCCCTATACATTGTTCATTAGATCTTCGCTAACTCCAACCAGCTACTGGCAACTCCTCT
ACGCCTTCGAATCCTCCAGCGGCAGCATCAGCAACCTCTACAACATGATCAAATCCGTC
GCAAGCGACTGCTCCGATCCGACACTACTCGGCAACTTCATCGAAAACCACGACAATC
CCCGTTTCGCCTCGTATGTCCCACCCCCTCCCCTCCCTACAATCACACTCACTAATACAT
CTAACAGCTACACCTCCGACTACTCGCAAGCCAAAAACGTCCTCAGCTACATCTTCCTC
TCCGACGGCATCCCCATCGTCTACGCCGGCGAAGAACAGCACTACTCCGGCGGCAAGG
TGCCCTACAACCGCGAAGCGACCTGGCTTTCAGGCTACGACACCTCCGCAGAGCTGTA
CACCTGGATAGCCACCACGAACGCGATCCGCAAACTAGCCATCTCAGCTGACTCGGCC
TACATTACCTACGCGGTTCGTCCTTCCCTCCCACCCTTTACCCCCCACCCTACAAACATC
CCACATACTAACAACATTTCAATAATGAAATAGAATGATGCATTCTACACTGACAGCAA

CACCATCGCAATGCGCAAAGGCACCTCAGGGAGCCAAGTCATCACCGTCCTCTCCAAC
AAAGGCTCCTCAGGAAGCAGCTACACCCTGACCCTCAGCGGAAGCGGCTACACATCC
GGCACGAAGCTGATCGAAGCGTACACATGCACATCCGTGACCGTGGACTCGAGCGGC
GATATTCCCGTGCCGATGGCGTCGGGATTACCGAGAGTTCTTCTGCCCGCGTCCGTCGT
CGATAGCTCTTCGCTCTGTGGCGGGAGCGGAAGATTATACGTCGAGTAA

3. The sequences of *pyrG* sgRNA expression cassette

Green: *A. fumigatus* U6 promoter

Black: linker

Orange: *pyrG* protospacer

Blue: sgRNA scaffold

Red: *A. oryzae* U6 terminator

gcaggcggttgcaagcgatctatcgtgaatttgtggagggacgtaatggtgaaatgccgaatggcgaagcaggtcgggagatcggtggtcg
gacgagcctgatgtagatgggtgcatatctttcttttctctttcacaatttcaagatcatgtattagaactacattgctatgtttcagcaatatatatgca
tagctagagagcgatgttacgatgagacatgtaatctttgaacatttctctgtacagcttgtcagagtttcgaaggttaagctcgagagcaagga
ggggaaaagaagacaaaaaagacactcggtccggtctcgaaccgacgacctttcgcacacacgtagaaggtacagaaactcaacacctcc
aaaagaggtctgtaagaatgaaagTAAACTCTTCTCCATCGCCGGTTTTAGAGCTAGAAATAGCAAG
TTAAAATAAGGCTAGTCCGTTATCAACTTGAAAAAGTGGCACCGAGTCGGTGCTTTTTT
TTTGAGCATTTATCAGCTTGATATAGAGGTAGGAATGTATGGAGGTGCAGAATGGCTATT
TTGTTATTGGAGCGGGTTCGAAACGGAGGGCAGGAGACTTTTTCTAAATACGTCACGT
GATATAGAGCTGCT

Table S5 The T7 transcription system of the protospacer RNA.

T7- protospacer- sgRNA scaffold fragment	10 μL
T7 transcriptase	2 μL
Buffer	10 μL
RNase free water	8 μL
Total	30 μL

37℃，16 h。

Table S6 The ethanol precipitation system of the RNA.

sgRNA product	30 μL
Isopropyl alcohol	300 μL
RNase free water	120 μL
Total	450 μL

-20℃， 2 h

Table S7 The NLS-Cas9 Nuclease cleavage of the target gene system in vitro.

NLS-Cas9 Nuclease	1 μL
Buffer	2 μL
Target gene DNA fragment	5 μL
sgRNA product	2 μL
RNase free water	10 μL
Total	20 μL

37℃，6 h or overnight

Table S8 The quality control of the RNA samples.

Name of sample	Concentration (ng/μL)	OD260/280	OD260/230	RIN/RQN	28S/18S	Result
G1	325	2.15	2.46	9.4	2.4	Qualified
G2	452	2.15	2.26	9.5	2.3	Qualified
N1	395	2.14	2.25	9.4	2.1	Qualified
N2	412	2.17	2.13	9.4	2.1	Qualified

Table S9 The expression of conidia-associated genes (CAGs) in the spore-like propagule and normal hypha of *Aspergillus niger* SH2.

Gene ID	Gene name/PFAMs	N FPKM	G FPKM	$\log_2$(N/G)	*P*-value
An12g10720	*Cat3*	699.69	13.31	5.14	0.0000
An12g10710	Hemerythrin	330.76	16.47	3.75	0.0000
An03g02400	*HfbA*	17728.19	836.07	4.41	0.0000
An08g09880	*HfbD*	22884.13	0.00	16.66	0.0000
An15g07300	AAA_22,NB-ARC	606.17	2.69	7.24	0.0000
An17g01885	CHZ	1962.83	31.48	5.38	0.0000
An12g10240	*ConJ*	883.11	30.08	4.29	0.0000
An15g04670	DUF1295	761.49	9.60	5.73	0.0000
An14g04530	NmrA	398.41	84.82	1.65	0.0000
An03g00920	Epimerase	143.36	0.41	7.79	0.0000
An01g10950	DJ-1_PfpI,ThiJ_like	155.42	18.93	2.46	0.0000
An03g02190	*Hxt5*	213.22	15.65	3.19	0.0000
An15g04770	Pyridoxal_deC	117.28	10.72	2.87	0.0000
An09g03130	*CatA*	574.11	14.38	4.74	0.0000
An18g04120	-	579.79	2.91	6.99	0.0000
An11g08160	DUF2235	77.44	2.41	4.41	0.0000
An03g04860	*Nce102*	349.78	74.09	1.66	0.0000
An15g05990	adh_short_C2	204.54	50.12	1.45	0.0000
An09g06270	-	341.26	5.09	5.48	0.0000
An08g06600	-	178.92	3.42	5.11	0.0000
An01g00280	HAD	107.11	9.28	2.95	0.0000
An02g07350	-	1221.27	159.91	2.35	0.0000
An09g05520	-	228.88	18.76	3.03	0.0000
An11g01750	*PilB*	101.60	9.01	2.92	0.0000
An02g08740	DUF3292	122.42	5.11	4.00	0.0000
An14g06050	-	683.00	135.41	1.76	0.0000
An08g03030	Lactamase_B_2	53.92	0.75	5.54	0.0000
An08g06620	-	1481.00	46.47	4.41	0.0000
An09g01150	adh_short	292.26	20.23	3.28	0.0000
An16g04420	*Ish1*	436.62	146.62	1.00	0.0000
An11g06120	*GndB*	57.25	2.22	4.10	0.0000
An04g04280	DUF1772	260.04	21.23	3.03	0.0000
An04g00100	DUF1761	2478.41	649.49	1.35	0.0000
An18g01860	*Fhk1*	55.55	9.47	1.97	0.0000
An08g08500	DUF3140,Med22	80.69	2.07	4.65	0.0000
An07g03930	Ferritin_2	512.07	6.67	5.68	0.0000
An04g05790	*VosA*	79.66	5.85	3.18	0.0000
An14g07380	ADH_zinc_N_2	14.37	0.58	3.98	0.0013
An01g05320	Abhydrolase	229.33	34.01	2.18	0.0000

An11g02200	*HppD*	217.06	21.50	2.76	0.0000
An12g01460	Bac_rhodopsin	556.33	30.36	3.61	0.0000
An04g09030	-*Sfc1*	187.97	18.01	2.80	0.0000
An16g05030	-	1927.62	162.99	2.99	0.0000
An07g06530	*Sur7*	711.33	32.00	3.89	0.0000

Table S10 The expression of germination-associated genes (GeAGs) in the spore-like propagule and normal hypha.

Gene ID	Gene name	N FPKM	G FPKM	log_2(N/G)	*P*-value
An16g03330	*Bys1*	2098.74	802.17	0.81	0.0000
An04g02550	*Mef1*	70.08	34.82	0.43	0.1667
An04g02000	*Ytm1*	39.35	15.84	0.74	0.1139
An12g08230	*Mrs2*	20.86	32.92	-0.66	0.0121
An01g10790	S	15149.26	3.60	12.04	0.0000
An08g03290	*Nop52*	28.58	12.26	0.64	0.2106
An01g03230	SRP40_C	41.69	30.96	0.43	0.7890
An01g04590	*Nat10*	30.89	15.94	0.38	0.4410
An16g04970	*Cpa2*	127.01	94.55	0.43	0.6514
An04g01900	*Utp13*	33.57	8.40	1.42	0.0118
An15g06390	*Rrp5*	30.38	14.84	0.46	0.3512
An16g05290	*Fcy21*	0.60	1.66	-1.48	0.3644
An13g01230	zf-LYAR	40.33	60.69	-0.59	0.0010
An09g06310	*Rrp15*	24.56	9.38	0.81	0.1537
An04g00680	*Nop15*	89.00	43.08	0.47	0.1030
An11g10000	*Ipi3*	16.88	5.13	1.13	0.1695
An07g06640	*Grc3*	14.51	3.22	1.59	0.1027
An11g06110	*Meu1*	116.12	64.49	0.27	0.2484
An14g01560	*Zuo1*	190.47	146.52	0.38	0.4209
An08g09160	*Utp10*	38.73	17.46	0.57	0.1915
An02g14340	*Snu13*	347.87	272.73	0.35	0.1931
An02g09200	*Rps0*	690.43	329.60	0.49	0.0000
An01g14080	*Rps22*	891.54	686.11	0.38	0.0556
An04g09270	*Utp8*	42.76	18.71	0.62	0.1706
An02g03860	Rrp12	37.37	19.24	0.38	0.3411
An18g04470	*Tbf1*	22.59	3.745	1.99	0.1027
An17g01270	*Mrpl7*	100.17	54.16	0.31	0.2409
An07g06760	*Rps20*	1230.21	797.74	0.62	0.2337
An15g01160	*Ecm16*	13.83	4.82	0.94	0.2966
An18g04220	*Pet9*	2450.64	2298.74	0.09	0.0000
An08g03910	FMO-like,Pyr_redox_3	1434.63	1198.21	0.26	0.0000
An16g07400	*Mtd1*	109.15	68.72	1.59	0.5989
An02g06530	*Clu1*	79.01	72.43	0.13	0.1506
An02g07010	*Ubi3*	1437.33	1151.16	0.32	0.0014
An02g08080	*Rpl22*	613.64	555.79	0.14	0.0000
An14g06860	*Oac1*	75.65	51.19	0.56	0.9174
An11g09740	*Ipi3*	26.22	7.24	1.28	0.0562
An11g11150	*Utp22*	22.06	6.36	1.22	0.0862
An11g00990	*Lag1*	86.14	31.09	0.89	0.0076

An11g05510	*Fpr3*	167.48	115.35	0.54	0.9981
An02g06320	*Erg13*	237.14	87.53	0.86	0.0000
An01g08850	*Cpc2*	1023.66	583.26	0.23	0.0009
An09g03120	GPI-anchored	67.62	13.47	5.02	0.0000
An11g06810	*Mrpl6*	58.94	42.11	0.49	0.9043
An07g07840	*Rpl23A*	659.48	424.41	0.64	0.3578

Table S11 The expression of genes involved in the production of enzymes involved in cell wall synthesis or processing.

Gene ID	Gene name	N FPKM	G FPKM	log_2(N/G)	*P*-value
An14g05350	*Ayg1*	501.47	0.60	9.71	0.0000
An09g05730	*FwnA*	386.86	6.00	6.01	0.0000
An03g03750	*McoC*	55.32	36.32	0.61	0.8422
An03g02360	*HfbB*	16927.17	451.67	5.23	0.0000
An04g08500	Hydrophobin	208.53	11.41	4.19	0.0000
An12g05020	*HfbE*	63.72	207.81	-1.71	0.0000
An07g03340	*Hyp1*	150.61	22.83	2.72	0.0000
An09g06400	*CtcA*	14.82	13.11	0.18	0.6398
An04g01430	SAP	786.88	1024.45	-0.38	0.0000
An06g01000	CFEM	221.77	937.58	-2.08	0.0000
An07g05570	*ChsA*	53.69	55.22	-0.04	0.0849
An09g04010	*ChsB*	158.37	107.73	0.56	0.9117
An12g10380	*ChsF*	28.14	8.41	1.74	0.0065
An02g02340	*ChsL*	42.62	140.55	-1.72	0.0000
An02g02360	*ChsM*	24.41	89.49	-1.87	0.0000
An09g02290	ChsD	25.94	27.15	-0.06	0.2171
An08g10740	DLH	109.61	140.01	-0.35	0.0170
An12g09130	DLH	171.55	86.32	0.99	0.0000
An06g01550	*FksA*	142.07	106.305	0.42	0.0491
An17g02120	*Smi1*	84.49	104.34	-0.30	0.5972
An09g03070	*AgsE*	90.61	39.04	1.21	0.0011
An04g09890	*AgsA*	7.37	6.34	0.22	0.0001
An11g07660	*ExgD*	21.16	10.04	1.07	0.6912
An03g05290	*BgtB*	1027.95	549.34	0.90	0.4602
An07g04650	*BgtC*	151.93	54.40	1.48	0.0000
An19g00090	LysM,Pectate_lyase_3	1.52	1.39	0.13	0.0003
An16g06800	*EglB*	74.52	45.39	0.72	0.8607
An03g06220	Glyco_hydro_72,X8	55.86	6.01	3.22	0.5830
An16g02850	Glyco_hydro_16	27.78	13.53	1.04	0.0000
An01g11010	*CrhD*	120.85	43.99	1.46	0.3482
An07g01160	*CrhC*	4.14	29.43	-2.83	0.0017
An07g07530	*CrhB*	38.38	43.09	-0.17	0.0690
An08g03580	*Bgt1*	2301.31	7.81	8.20	0.0000
An10g00400	*GelA*	532.66	403.66	0.40	0.2242
An16g07040	*BtgE*	26.48	67.87	-1.36	0.0002

Table S12 The expression and spacer RNA sites of genes involved in the N-acetyl-D-glucosamine metabolic pathway and conidia.

Gene ID	Name	$Log_2(N/G)$	Spacer RNA sequence (5'-3')	Description
An16g09020	*Ngt1*	2.22	GTATGTGC AGGTGTAA ACAG	N-acetylglucosamine transmembrane transporter activity
An13g00510	*Hxk1*	3.23	GTGCAAG ACCTTGAC CAATG	Hexokinase
An16g09040	*Dac1*	1.96	GAAGTGTC TGAACTAC TAAG	N-acetylglucosamine-6-pho sphate deacetylase activity
An12g00480	*Uap1*	0.12	ACAAGGG TGTGAAAG CGCGG	UDP-N-acetylglucosamine pyrophosphorylase
An08g09880	*HfbD*	16.66	GGATGATA GAAAACTT CAGG	expressed in dormant conidia
An01g10790	GC	12.03	GGTGACAT TATGACTT CATG	expressed in germinating conidia
An06g02240	H-MAPK	14.58	CCCTGTTC ATTGCTCC AAAA	MAPK signaling pathway

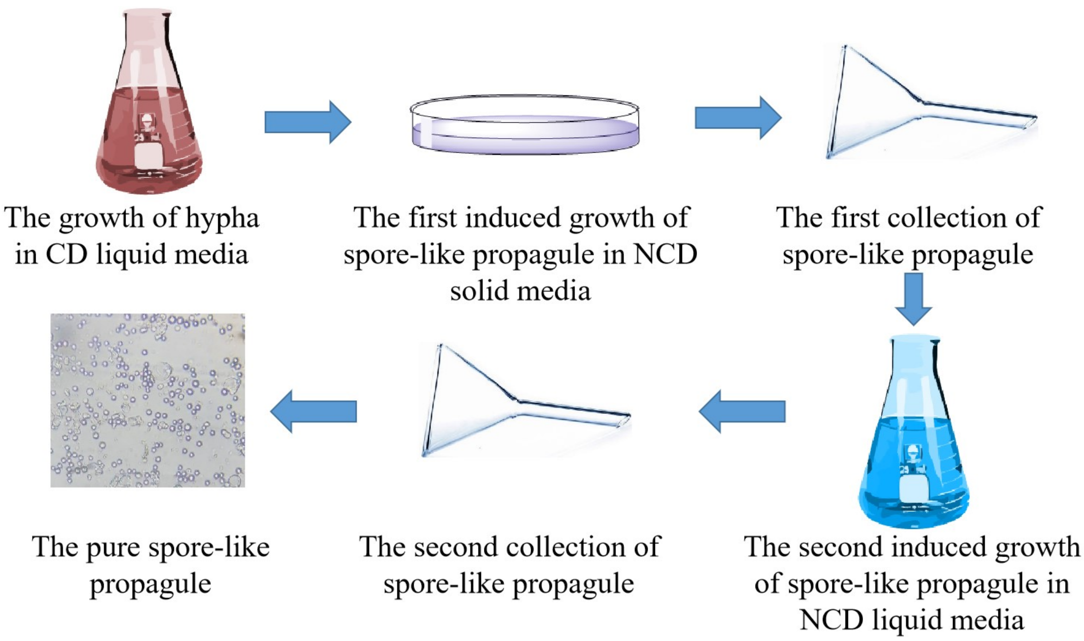

Figure S1 The flowchart of spore-like propagule purification.

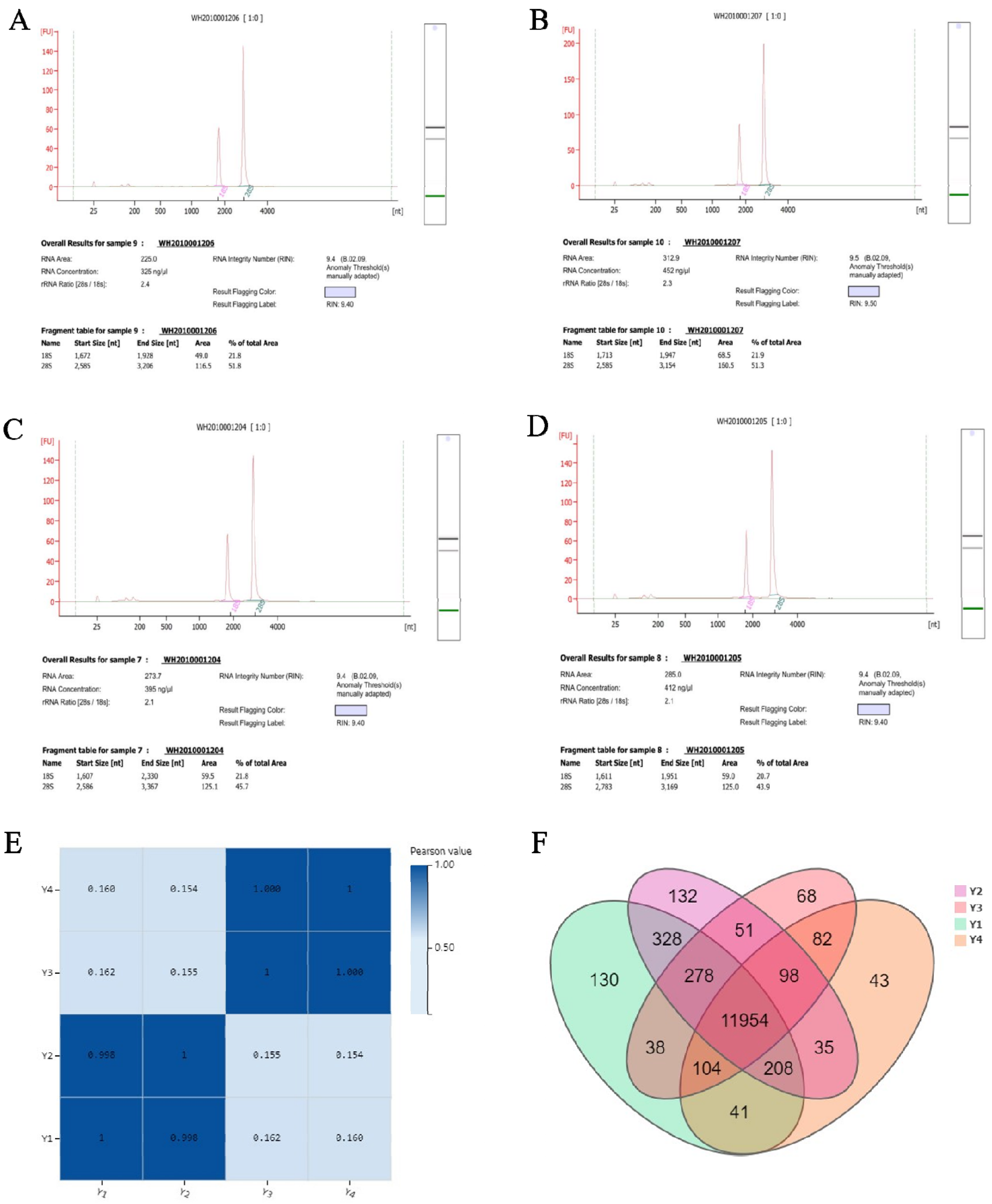

Figure S2 The RNA sequence of spore-like propagule. (A) The result of sample Y-1 (N1) RNA quality was assessed with Agilent 2100. (B) The result of sample Y-2 (N2) RNA quality was assessed with Agilent 2100. (C) The result of sample Y-3 (G1) RNA quality was assessed with Agilent 2100. (D) The result of sample Y-4 (G2) RNA quality was assessed with Agilent 2100. (E) The heat map of correlation between the samples. (F) The venn diagram of expression level between the samples.

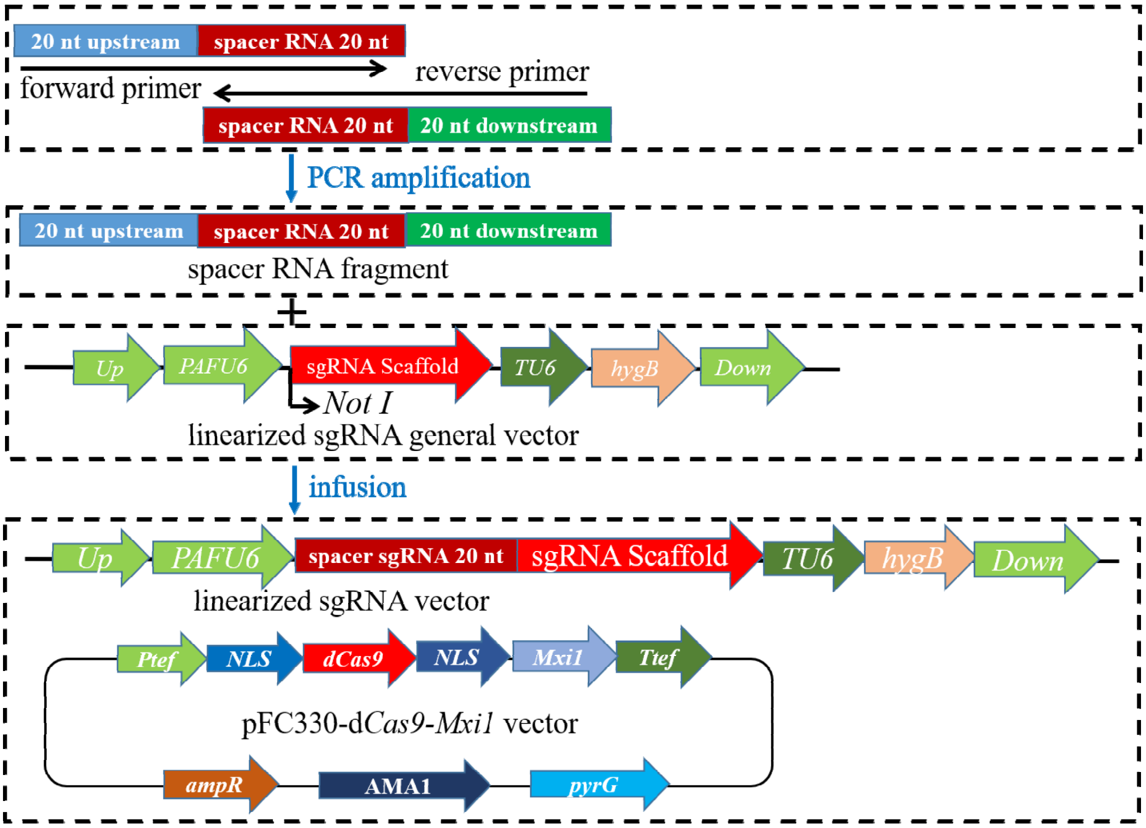

Figure S3 The flow chart of CRISPRi construction on conidia and GlcNAc metabolic pathway genes. The short spacer RNA fragment (total 60 bp) with 20 bp upstream homologous arm, 20 bp spacer and 20 bp upstream homologous arm was obtained by PCR amplification without template DNA. And the short spacer RNA fragment was connected to linearized sgRNA general vector using infusion method. The sgRNA general vector was linearized by *Not1* enzyme.

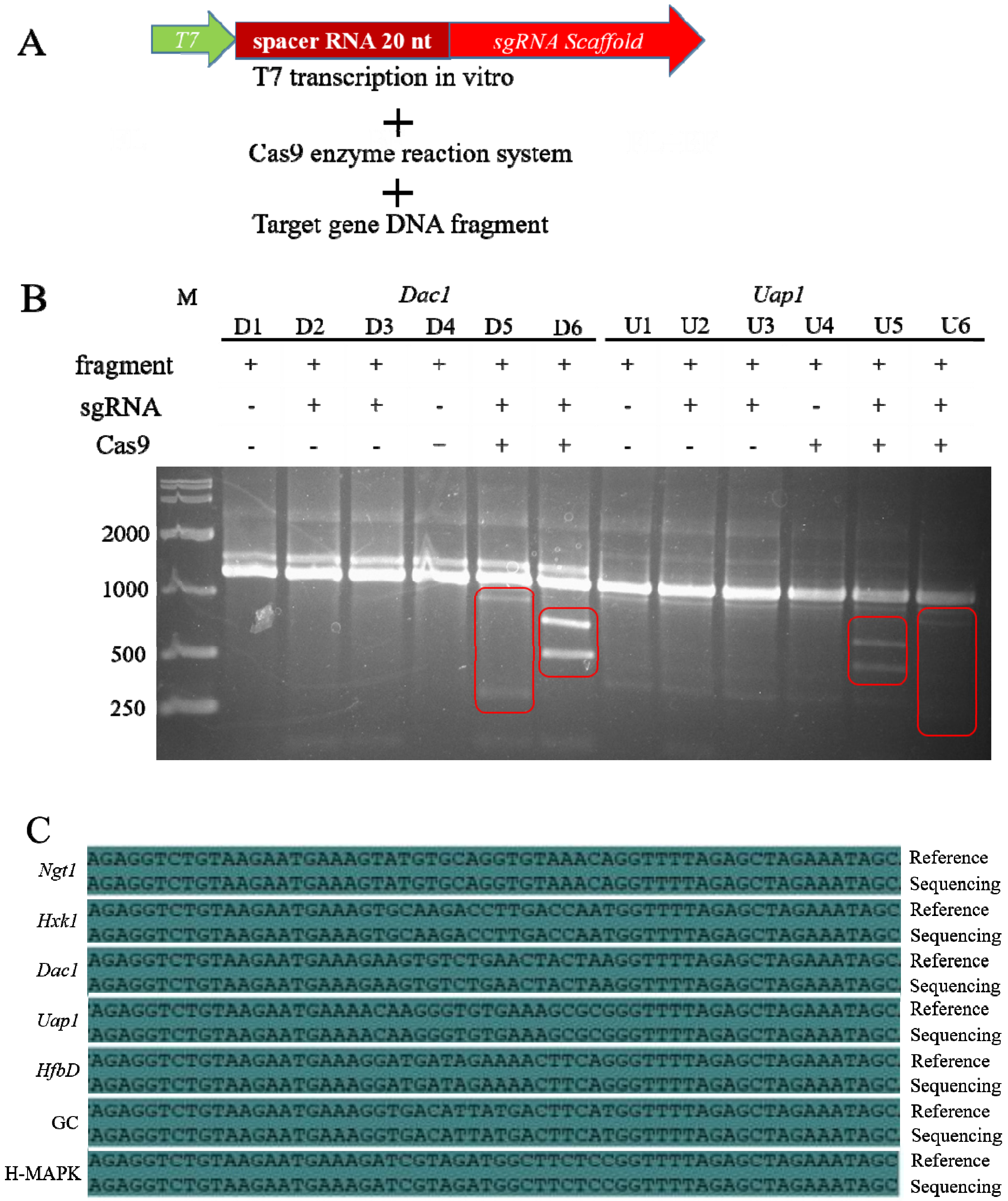

Figure S4 The construction of CRISPRi on conidia and GlcNAc metabolic pathway genes in the *Aspergillus niger* SH2. (A) The T7 transcription of sgRNA in vitro. (B) The Cas9 digestion of a 1200 bp DNA fragment with each sgRNA variant in vitro. (C) The sequencing and alignment of spacer RNA sequence that was connected into linearized sgRNA general vector using infusion method.
